# An Experimental Method for Fatigue Testing Cast Iron Water Pipes Using Combined Internal Water Pressure and Bending Loads

**DOI:** 10.1007/s11340-025-01153-6

**Published:** 2025-02-10

**Authors:** E. D. A. John, J. B. Boxall, R. P. Collins, E. T. Bowman, L. Susmel

**Affiliations:** 1 School of Mechanical, Aerospace and Civil Engineering, Sir Frederick Mappin Building, The University of Sheffield, Mappin Street, Sheffield, S1 4DT UK; 2 Materials and Engineering Research Institute, Sheffield Hallam University, Harmer Building, Sheffield, S1 1WB UK

**Keywords:** Biaxial fatigue, Water pipe, Grey cast iron, Constant amplitude, Experiment verification

## Abstract

**Background:**

Investigations into the fatigue failures mechanism of Grey Cast Iron (GCI) water pipes are inhibited by the lack of a lab-based method to conduct extensive high-cycle biaxial fatigue test programmes.

**Objective:**

The work presented in this paper developed and tested a novel experiment capable of causing controlled fatigue failures of GCI pipe specimens in the high-cycle fatigue regime using bending and internal water pressure fatigue loading.

**Methods:**

A novel four-point bending and internal water pressure fatigue testing system was developed to apply constant amplitude out-of-phase biaxial loading to 58 mm diameter GCI pipes at 1.7 Hz. To verify the ability of this equipment to apply known stresses and repeatable loads to pipe specimens a series of tests were conducted. A finite element model of the pipe specimen was used to estimate the strains and displacements applied by the equipment.

**Results:**

Experimental strains and displacements were mainly within ± 10% of the estimated values and the pressure amplitudes measured over 10^3^ cycles were within ± 3% of the average. Dynamic load effects occurred at higher bending loads, but these were quantified and accounted for. Trial destructive tests revealed that the lifespan of leaking fatigue cracks in GCI pipes with uniform wall-loss subject to combined internal pressure and bending fatigue loads is less than 1% of the total cycles-to-burst.

**Conclusions:**

The experimental method developed was able to apply combined, out-of-phase internal pressure and bending fatigue loads accurately and consistently to small-dimeter GCI pipes, and cause these pipes to develop high-cycle fatigue regime failures.

## Introduction

Reducing and preventing leakage and pipe breaks to improve system resilience is a priority of drinking water distribution network managers in many countries, including the UK and USA [1, 2]. Understanding and modelling the mechanisms that cause pipes to fail will enable proactive replacement of water pipes before they reach a “failure” state, whether that be leak or burst. Grey Cast Iron (GCI) pipes are amongst the oldest water pipes still in service, having been installed pre-1960 in the UK, and are still common in many water distribution networks [2, 3]. GCI pipes can begin to leak due to the formation through-wall cracks at stress-concentrating corrosion pits [4, 5].

The mechanical properties of GCI pipes, such as their elastic modulus, tensile strength, and fatigue strength, are known to vary significantly between pipes, and even between material samples taken from the same pipe (see John et al. [6] and the references therein). For example, elastic moduli ranging between 76 and 178 GPa are reported for exhumed GCI pipes [7, 8]. This variation is understood to result from differences in the casting and cooling process between pipes, and within a single pipe, and the random distribution of inclusions, such as graphite flakes, within the material’s microstructure [9].

Smaller diameter GCI pipes can experience biaxial stress states; internal water pressure causes a pipe to experience stress acting around its circumference [10, 11] and bending loads, such as vehicle weight and soil moisture response, may cause stress acting in the pipe’s axial direction [11, 12]. These loads are also time variable, and some can cycle tens of times per day [13, 14]. Seica and Packer [15] found that the higher compressive strength of GCI pipes, relative to their tensile strength, meant that static failure analysis of these pipes subject to bending loads required non-linear analysis techniques.

The formation of fatigue cracks in corroded GCI pipes has been proposed as a potential leak initiation mechanism, with corrosion pitting acting as a notch that amplifies the fatigue damage caused [16, 17]. Although previous authors have investigated the notch fatigue strength of GCI specimens featuring sharp circumferential notches [18, 19], the fatigue reduction effect of localised pit-like notches in GCI pipes subjected to biaxial loading is currently unknown. Without suitable fatigue test data it is not possible to develop a validated multiaxial notch fatigue model for GCI water pipes. Furthermore, the nature of leaks formed in these conditions are poorly understood, with only a small number of tests leaking cracks formed under ramped pressure loads reported in the literature [20, 21].

Controlled, repeatable laboratory tests are required to confirm that fatigue loading can cause GCI water pipes to develop leaking through-wall fatigue cracks, and to validate a fatigue failure criterion that can account for multiaxial loading and the notch effect of corrosion pits. The Smith–Watson–Topper multiaxial fatigue criterion, validated by John et al. [22] for un-notched water pipe GCI, predicts that 180° out-of-phase biaxial fatigue stresses are more damaging to GCI pipe material than in-phase biaxial or uniaxial fatigue stresses. Therefore, laboratory tests of notched GCI water pipes must include cyclic, out-of-phase biaxial stress conditions to investigate the effect of complex loading on the time taken for a leaking crack to form.

Brevis et al. [16] and Jiang et al. [17] predicted that around 10^4^ to 10^6^ load cycles occurring over the final years of a pipe’s life would be required to cause fatigue failure, meaning these failures can be classed as high-cycle fatigue (failures occurring between 10^2^ and 10^6^ cycles for GCI) [23]. Previously, Rathnayaka et al. [20] developed an internal pressure fatigue test facility for large diameter GCI pipes that was able to apply around 2,000 pressure cycles per day. Other authors have also reported fatigue tests of non-GCI pipe sections using internal pressure fatigue loads or bending fatigue loads, either independently or in-phase [24–28]. However, no process or equipment has previously been developed that can apply out-of-phase biaxial fatigue stresses to a GCI water pipe at a frequency that enables large programmes of tests lasting around 10^4^ cycles.

To enable extensive investigations into the fatigue failure mechanism of GCI water pipes for the first time, the work presented in this paper aimed to develop and test a novel experiment capable of causing controlled fatigue failures of GCI pipe specimens in the high-cycle fatigue regime using combined bending and internal water pressure fatigue loading. This paper details the design, verification, and testing of this experiment. To confirm that the loads applied by the experiment resulted in the pipe specimens experiencing the intended stresses, measured strains and displacements were compared with analytical and Finite Element Analysis (FEA) estimations. To verify that dynamic load effects did not influence the fatigue loads applied by the experiment, the dynamic behaviour of each load was characterised. To assess the cyclic accuracy of the amplitude and phasing of the applied loads, several trial tests were run for 10^3^ load cycles and the applied loads were recorded. As mentioned above, 180° out-of-phase equibiaxial loading is expected to be more damaging to GCI than in-phase equibiaxial loading, so 180° out-of-phase loading was prioritised for this testing. To confirm that the experiment was able to cause fatigue failure of the GCI pipe specimens, three destructive tests were run with combined internal pressure and bending fatigue loading.

## Methods

### Experiment Design

This section details the design of the novel internal water pressure and bending fatigue experiment developed as part of this work. Following an explanation of the maximum design loads selected for the experiment, the following key aspects of the design are discussed: the GCI pipe specimens, bending load application, internal water pressure application, loading control, and instrumentation.

A key aspect of the experiment design was the magnitude of the bending and internal water pressure loads. So that the stresses experienced by pipe specimens could be adjusted to cause high-cycle fatigue regime failures in any test scenario, the experiment was designed to apply a wide range of load magnitudes. To ensure that the experiment could cause high-cycle fatigue regime failures of any GCI pipe specimens, including as-new specimens, very high maximum design loads were selected (20 kN bending load and 20 MPa internal pressure load). Fatigue tests conducted under higher, or lower, loads than those experienced by in-service water pipes can be translated to real loads by considering the stresses experienced by the pipe material.

#### Pipe Specimens

To investigate the effect of internal water pressure and bending fatigue loading, and the resultant pipe failure mode, sections of complete pipe barrel were tested. So that tests would be repeatable, nominally-identical GCI material that was free from “real” corrosion was used for all tests.

**Fig. 1:**
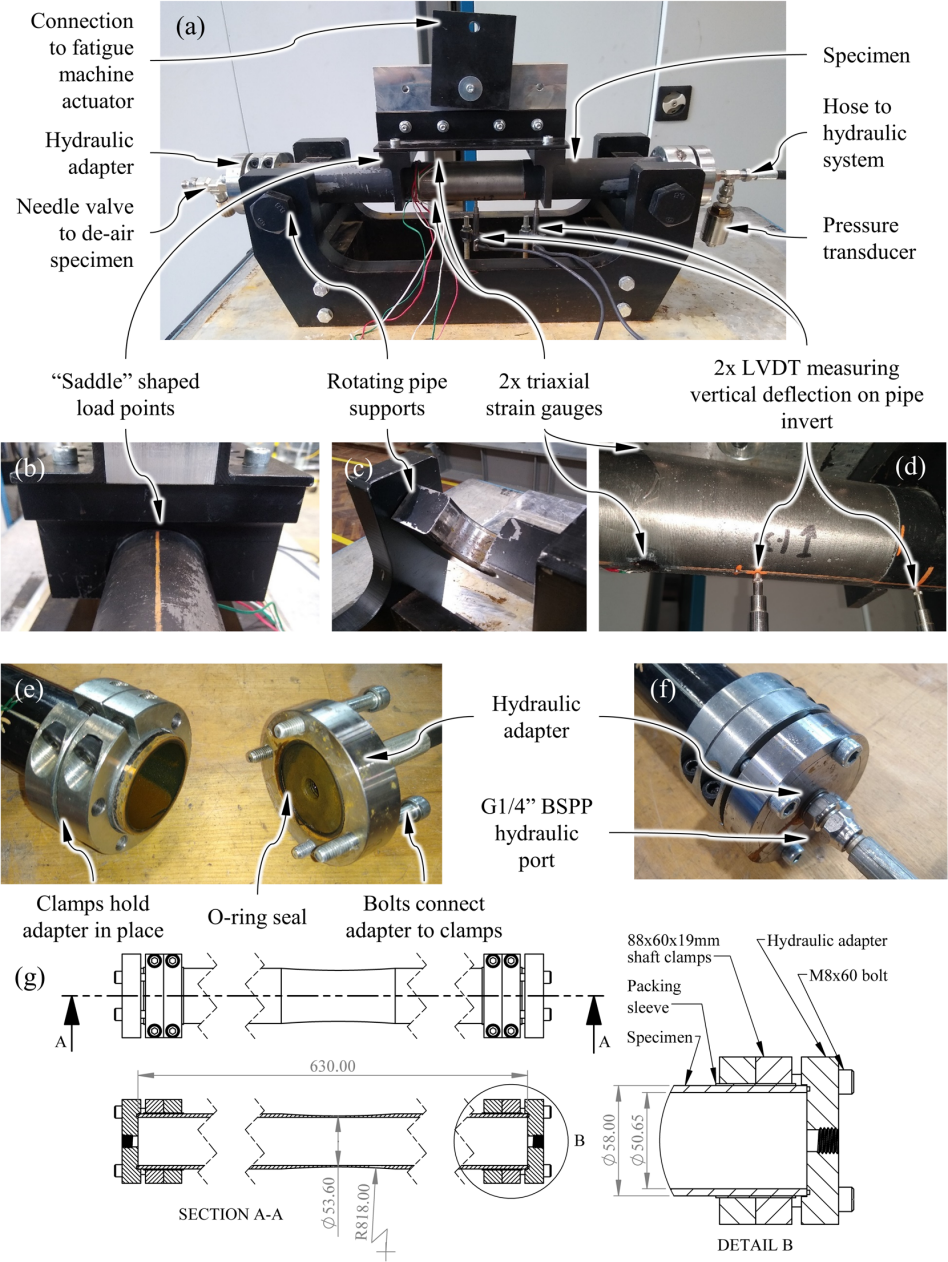
Labelled photographs and drawings showing the four-point bending apparatus, pipe specimens, and pipe specimen hydraulic adapters

Obtaining multiple intact, exhumed pipe barrels that were un-corroded and with nominally-identical material properties was not possible. Instead, test specimens were produced from new spun-cast GCI soil pipes manufactured in accordance with BS 416–2 [29]. Previous work by the current authors confirmed that new BS 416–2 pipes have very similar tensile and fatigue properties to exhumed GCI water pipes [6, 22]. BS 416–2 pipes were available with nominal internal diameters from 50 to 150 mm. The focus of the experiment was on the material fatigue response to applied stresses, so there was no need to reproduce exact water pipe dimensions. From an experimental perspective, a small pipe diameter was preferred as this facilitated faster test set-up and execution as smaller dimeter pipes are less cumbersome and required lower loads to fail, enabling higher loading frequencies. Therefore, BS 416–2 pipes with a 50 mm nominal internal diameter, sourced from a single manufacturer, were used as test specimens (see Fig. 1(a)). Pipes were sourced from a single manufacturer in an effort to reduce the difference in material properties between specimens. The as-supplied external diameters of these pipes were about 58 mm, and the internal diameters were about 51 mm. The smallest in-service GCI water pipes in the UK have external diameters around 96 mm and internal diameters around 81 mm [30], so the dimensions of the pipe specimens tested were of a similar magnitude to in-service pipes.

To apply internal water pressure loading to the pipe specimens, adaptors were developed that allowed specimens to be connected to the internal water pressure loading system detailed below, and allowed pressurised water to be contained within the specimen. To contain the pressurised water in a way that did not interfere with the application of bending loading, end caps were fitted to each specimen. These end caps converted the pipe specimens into closed-ended pressure vessels and functioned as hydraulic adapters, as shown by Fig. 1(a) and (f). The hydraulic adapters were bolted to a pair of shaft clamps, as shown by Fig. 1(e) and (g), which were secured to the pipe specimen by the friction resulting from tightening the shaft clamp bolts to 30 Nm. Consequently, the pressure reaction force on each of these end caps was transmitted directly to the pipe specimens as an axial stress.

#### Bending Loading

To create a region of constant maximum bending stress in the pipe specimens away from the stress-concentrating influence of the load points a four-point bending arrangement was used. The design of the supports and load points was developed to cause conditions close to an ideal, simply supported, four-point bending case.

Deformation of the specimen cross-section was minimised by using saddle shaped load points and pipe supports, as shown by Fig. 1(b) and (c). Preliminary calculations estimated that the axial movement of the specimen's geometric centroid at the support locations would be extremely low. This enabled the roller support aspect of the classical simply supported arrangement to be removed, and instead the specimen supports were mounted on pivots in-line with the specimen centroid, as shown by Fig. 1(a) and (c).

**Fig. 2:**
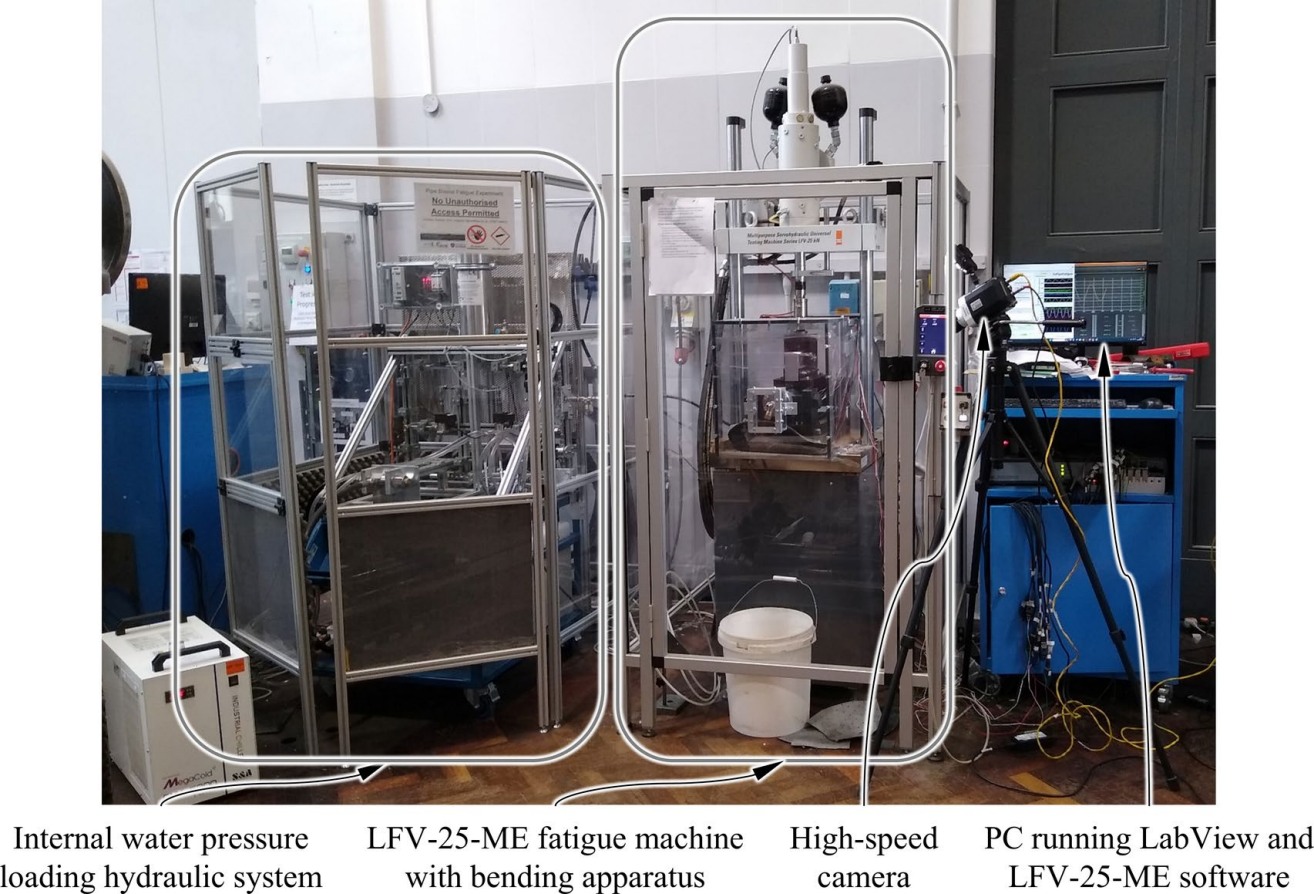
Labelled photograph showing the full experimental set-up

A load spacing of 200 mm and support spacing of 480 mm were used and loading was applied by a walter + bai ag LVF-25-ME servo-hydraulic fatigue testing system with a maximum fatigue load of ± 20 kN. The complete bending fatigue arrangement is shown in Fig. 2. The bending apparatus was designed to test pipes up to 100 mm in diameter, but this would require new supports and load points to be fitted.

#### Internal Water Pressure Loading

The internal water pressure loading system was designed to cause fatigue failure of the pipe specimens detailed above using an alternating, constant amplitude internal water pressure load. The internal water pressure loading system was designed such that it could operate independently, or synchronously with the bending fatigue load.

To reflect the fact that water pressure loads experienced by in-service pipes tend to have mean pressures greater than zero the system was designed to apply pressure loads with a load ratio of 0.1. As discussed above, a very high maximum design pressure of 20 MPa was used for this system. To enable a wide range of possible internal water pressure amplitudes the system was designed to apply maximum cyclic pressures down to 3.5 MPa.

**Fig. 3:**
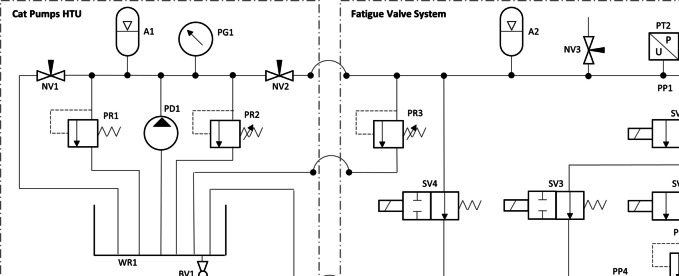
Full schematic for the hydraulic system used to apply internal water pressure fatigue loading to pipe specimens (see Table 1 for component details)

To enable fatigue tests of up to 10^5^ cycles to be completed within a few days (during working hours), the system was designed for a loading frequency of 2 Hz. So that this fast cycle frequency could be achieved, a pair of solenoid valves (Fig. 3, SV1 and SV2) were used to alternately expose the pipe specimen to a high-pressure reservoir (Fig. 3, A2) and low-pressure reservoir (Fig. 3, WR1) thereby cycling the pressure in the specimen between two controlled pressure levels. Using solenoid valves also enabled precise control of the pressure cycle timings. To reduce the risk of cavitation, the minimum cycle pressure was held above zero using a manually set pressure control valve on the hydraulic line to the low-pressure reservoir (Fig. 3, PR4). The maximum cyclic pressure could be set between 3.5 and 20.0 MPa (using PR2 and PR3, Fig. 3), while the minimum cyclic pressure could be set between 0.7 and 7.0 MPa, allowing a load ratio of 0.1 at all but the lowest pressures.

**Table 1:** Hydraulic system major components list. The “Ref. No.” column refers to Fig. 3 component labels

Ref. No	Description	Key specifications
A1	Nitrogen-charged accumulator, HTU	Working volume: 0.1 L Max. pressure: 21 MPa
A2	Nitrogen-charged accumulator, FVS	Working volume: 2 L Max. pressure: 25 MPa
BV1	Ball valve, WR1 drain	
NV1	Needle valve, HTU return line	
NV2	Needle valve, HTU outlet	
PD1	Positive displacement pump	Max. flowrate: 7.4 L/minute Max. pressure: 20 MPa
PG1	Pressure gauge	Pressure range: 0—40 MPa (gauge)
PG2	Pressure gauge	Pressure range: 0—40 MPa (gauge)
PP1-5	Pressure test port	
PR1	Pressure regulator, HTU relief valve	Max. flowrate: 25 L/minute Set pressure: 21 MPa
PR2	Pressure regulator, HTU primary	Max. flowrate: 3.8—38.0 L/minute Pressure range: 10.5—21.0 MPa
PR3	Pressure regulator, FVS step-down	Max. flowrate: 3.8—38.0 L/minute Pressure range: 3.5—14.0 MPa
PR4	Pressure regulator, FVS back pressure	Max. flowrate: 1.9—19.0 L/minute Pressure range: 0.7—70 MPa
PS1	Pipe specimen	
PT1-2	Pressure transducer	Max. pressure: 34.5 MPa (gauge)
SV1-2	Solenoid valve, pressure cycling	Normally closed 24 V DC Max. pressure: 25 MPa Orifice diameter: 6 mm
SV3-4	Solenoid valve, safety	Normally open 24 V DC Max. pressure: 30 MPa Orifice diameter: 0.5 mm
WR1	Water reservoir	Volume: 50 L

So that the stability of leaking fatigue cracks could be observed the system was designed to continue applying internal pressure loading after a small leak had formed. A pump was used to maintain pressure in the high-pressure reservoir before and after a leak formed (Fig. 3, PD1). This pump was supplied as part of a Hydrostatic Test Unit (HTU) as shown by Fig. 3. The high-pressure reservoir was a nitrogen-charged accumulator with a working volume of 2 L (Fig. 3, A2). The bespoke fatigue valve system (see Fig. 3) was mounted on the wheeled HTU frame and positioned within a safety enclosure next to the servo-hydraulic fatigue testing system (see Fig. 2).

#### Experiment Control and Instrumentation

Limitations of the LVF-25-ME controller meant that it could not be used to control the internal water pressure system solenoid valves, therefore two controllers were used. To control the timing of the solenoid valves a bespoke code for the software LabVIEW 2018 [31] was produced. When independent internal water pressure loading was required, the loading frequency was set using LabVIEW.

For synchronised bending and internal water pressure loading, the LVF-25-ME controller acted as the primary controller, independently applying a bending load at a set frequency. To synchronise the internal pressure load with the bending load LabVIEW received the signal of a Linear Variable Differential Transformer (LVDT) measuring the vertical displacement of the pipe specimen's invert at the midpoint. The LVDT signal was used to calculate the frequency and midpoint-crossing time of the bending load signal, from which the solenoid valve actuation timings for each cycle were determined. The phase difference between the two loads was set via LabVIEW. In practice, 2 Hz loading was achieved for internal pressure loading and 1.7 Hz was achieved for biaxial loading. The significant additional complexity in the biaxial loading control loop was the limiting factor for the biaxial loading frequency.

The bending load was measured using a load cell integral to the LFV-25-ME and the internal pressure load was measured at the inlet to the pipe specimen (as shown by Fig. 1(a)) using a gauge pressure transducer with a 34.5 MPa maximum pressure (RDP, PT-TJE-G05000). Vertical deflection was measured using LVDTs with a range of ± 2.5 mm (RDP, D5/100AWRA-L25), and strain measurements were made using 5 mm gauge length, 120 Ω triaxial strain gauge rosettes (Kyowa, KFGS-5–120-D17-11). To observe the onset of water loss a Phantom Miro M310 high-speed camera was used to record the expected location of crack initiation at 1,000 fps. The side-on position of the camera, shown by Fig. 2, meant a mirror positioned under the pipe specimen was used to record footage of the pipe invert during tests.

### Experiment Verification Tests

Verification of the bending and internal pressure fatigue experiment was required to give confidence in the results obtained from this new and novel experiment. The verification process aimed to assess if: the loads applied by the experiment resulted in the intended stresses in the pipe specimens; the fatigue loads applied by the experiment were influenced by dynamic load effects; the control system was able to accurately apply the loads over multiple cycles. For this verification testing, two randomly selected pipe specimens were used. To enable good strain gauge adhesion the gauge section of these specimens was turned to give a smooth surface (see the pipe specimen in Fig. 1(d)).

#### Static Load Tests

Validation experiments were performed for the three types of loading that the bending and internal pressure fatigue experiment was designed to apply: four-point bending, internal water pressure, and 180° out-of-phase bending and internal pressure. For the bending load verification and internal water pressure loading verification, loads equal to approximately half the full range of the experiment were applied to avoid damaging the specimens. Specifically, these were a 10 kN bending load and a 7.5 MPa internal water pressure load. To reflect the two extreme conditions of a 180° out-of-phase bending and internal pressure test, two different load conditions were tested. Firstly, a high bending load (15 kN) and low internal water pressure load (0.1 MPa) were tested, followed by a low bending load (1.5 kN) and high internal pressure load (7.5 MPa).

The uniformity of the axial stress along the specimen midsection was investigated by fitting three triaxial strain gauge rosettes along the pipe invert: one at the midpoint and one 40 mm away in each direction. Cross-section deformation of the specimen midpoint was measured by adding three additional triaxial strain gauge rosettes around the circumference of the pipe midpoint, so that there were rosettes at the invert, crown, and both springlines. To capture the deflection of the specimen, three LVDTs were positioned to measure vertical displacement of the specimen midpoint and beneath the load points (i.e. 100 mm either side of the midpoint).

#### Dynamic Load Tests

Trial bending fatigue tests revealed that the fatigue machine actuator had a travel range up to around 2 mm per load cycle making dynamic load effects a potential issue. To determine the extent of any dynamic load effects a process similar to that specified by ASTM E467-21 [32] and BS ISO 4965–1 [33] was used. To characterise the dynamic performance of the bending load system over the full range of load amplitudes and frequencies used in testing the following force amplitudes were tested at 1.7 Hz and 4.0 Hz with a load ratio of 0.1: 2.25 kN, 3.83 kN, 5.40 kN, 6.98 kN, and 8.55 kN. To estimate the true load applied to the pipe specimens under dynamic loading, strains measured under static and dynamic loading were compared. C_DCF_ was calculated for each combination of force amplitude and frequency using the equation given in BS ISO 4965–1 [33]:

1 $${C}_{DCF}=\frac{\Delta {F}_{dyn}}{\Delta {F}_{i}}$$

where: ΔF_dyn_ is the true dynamic force range; and ΔF_i_ is the indicated force range. To provide a continuous approximation of C_DCF_ that could be applied to any load amplitude, the value of C_DCF_ for each frequency-load combination was averaged and a quadratic curve was fitted to the data points for each frequency.

Specimen stiffness may also influence the dynamic behaviour of the system, so, in addition to the two 58 mm gauge diameter specimens a specimen with a reduced gauge section diameter of 54.5 mm was also tested at force amplitudes up to 6.3 kN. Higher force amplitudes risked damaging the reduced gauge section specimen.

Sudden valve actuation is known to be a source of high-frequency pressure transients in hydraulic systems. Pressure transients or instabilities with an amplitude approaching, or greater than, the intended pressure cycle amplitude would significantly alter the results of a fatigue test. To investigate whether any significant transient events occurred during an internal water pressure load cycle the water pressure at the pipe specimen was recorded at 10 kHz at the highest expected internal water pressure amplitude of 6.55 MPa.

#### Cyclic Loading Accuracy

The use of an open-loop system to control the minimum and maximum cyclic internal water pressure meant it was essential to verify the cyclic accuracy of this loading. Because the internal water pressure loading system was built for this study, rather than being a piece of commercial equipment, the cyclic load accuracy was assessed over 10^3^ cycles rather than the 50 cycles recommended by ASTM E467-21 [32] and BS ISO 4965–1 [33]. 10^3^ cycles reflected a realistic but short test duration. ASTM E467-21 [32] and BS ISO 4965–1 [33] both recommend that an error ≤  ± 1% of the target force range is acceptable. The use of manual pressure control valves meant a numerical target pressure value could not be specified; therefore, the measured minimum and maximum pressure value of each cycle were compared to the average measured minimum and maximum pressure values across all cycles.

The bespoke nature of the biaxial load phasing control system meant that verification of this system was also essential. The process used to determine the cyclic accuracy of the biaxial phasing controller and settings was similar to that detailed above for determining the cyclic load accuracy of the bending and internal pressure loads. The controller was only used to apply 180° out-of-phase loading where at one load's maximum value the other load would be at its minimum value, and vice versa. To verify the control system's accuracy when applying biaxial loading specimens were subject to 10^3^ cycles of 180° out-of-phase loading. For each load cycle, the pressure values concurrent with the minimum and maximum bending forces were identified. The accuracy of the controller was established by comparing these pressure values to the actual maximum and minimum pressure values of the cycle. Perfect agreement between the pressure values at the minimum and maximum forces and the actual cyclic maximum and minimum pressure values would correspond to perfect 180° out-of-phase loading.

### Analytical and FEA Strain and Displacement Estimations

To confirm that the loads applied by the experiment resulted in the pipe specimens experiencing the intended stresses, measured strains and displacements were compared with analytical and FEA estimations. Analytical strain and displacement for specimens subject to four-point bending were calculated using classical bending theory [34, 35]:

2 $${\varepsilon }_{x}=\frac{1}{E}\left(-\frac{{F}_{b}{a}_{b}y}{2I}\right)$$

3 $${\varepsilon }_{\theta }=\frac{1}{E}\left(\nu \frac{{F}_{b}{a}_{b}y}{2I}\right)$$

4 $$\delta y=\frac{1}{EI}\left(\frac{{F}_{b}{a}_{b}}{4}{x}^{2}-\frac{{F}_{b}{L}_{b}{a}_{b}}{4}x+\frac{{F}_{b}{a}_{b}^{3}}{12}\right)$$

where: ${\varepsilon }_{x}$ is axial strain; ${\varepsilon }_{\theta }$ is hoop strain; $\delta y$ is vertical deflection (negative in the direction of the applied force); $E$ is the material elastic modulus; $\nu$ is the material Poisson ratio (0.28 for this material); $I$ is the second moment of area of the beam cross-section; ${F}_{b}$ is the magnitude of the total applied bending force; ${a}_{b}$ is the distance along the beam between a support and the nearest load point; ${L}_{b}$ is the distance along the beam between the two supports; $y$ is the vertical distance from the beam centroid to the point at which strain is evaluated (negative in the direction of the applied force); and $x$ is the distance along the beam from one support at which deflection is calculated. Equation (4) is only valid between the two load points. Analytic strain estimations for pressure loading were made using the closed-end thick-walled pressure vessel relations [36]:

5 $${\varepsilon }_{x}=\frac{1-2\nu }{E}\left(\frac{{r}_{i}^{2}{P}_{i}}{{r}_{e}^{2}-{r}_{i}^{2}}\right)$$

6 $${\varepsilon }_{\theta }=\frac{2-\nu }{E}\left(\frac{{r}_{i}^{2}{P}_{i}}{{r}_{e}^{2}-{r}_{i}^{2}}\right)$$

where: ${P}_{i}$ is the internal pressure; ${r}_{i}$ is the pipe internal radius; and ${r}_{e}$ is the pipe external radius.

**Fig. 4:**
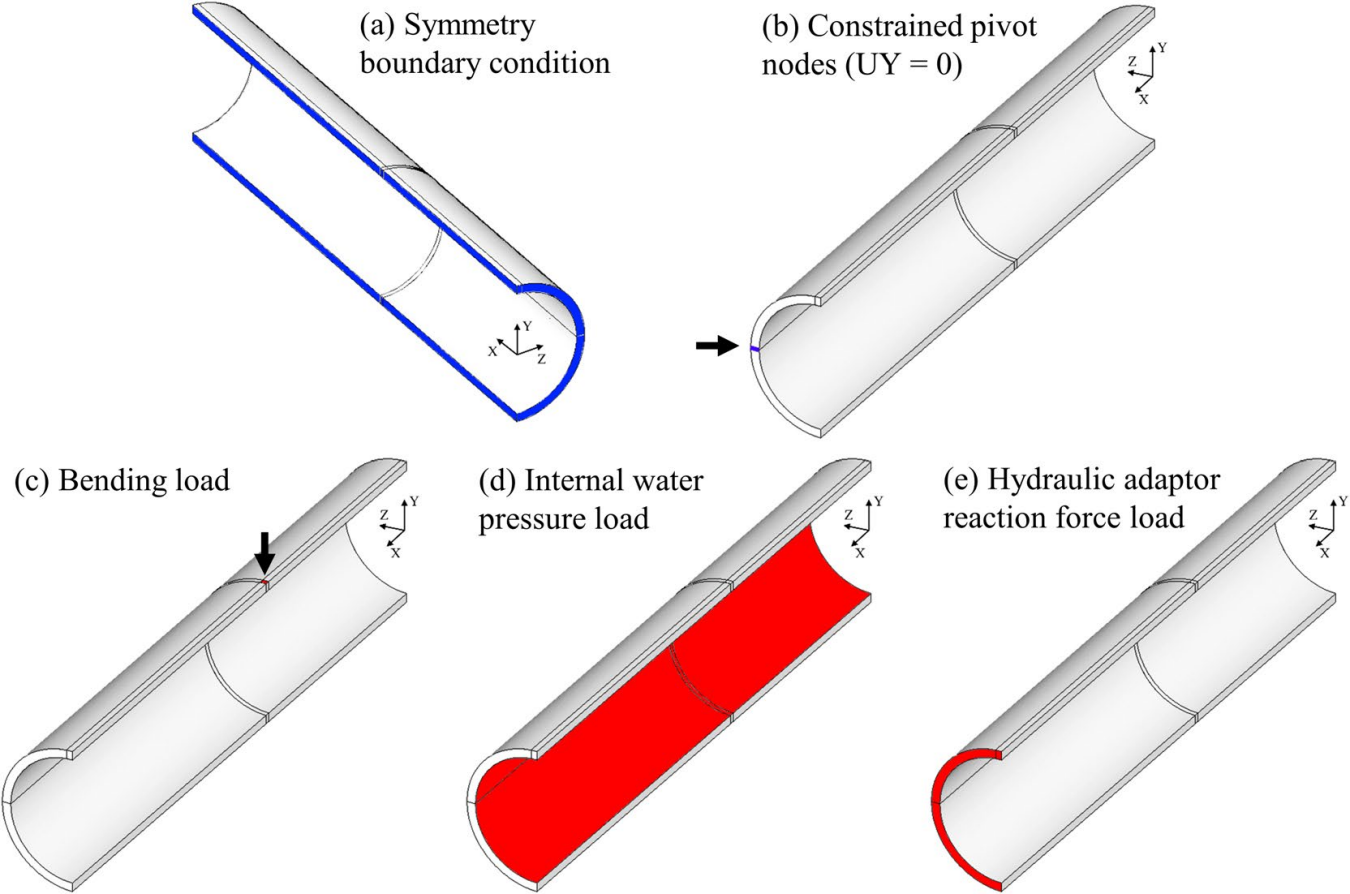
Diagrams showing the boundary conditions (shaded blue) and loads (shaded red) applied to the four-point bending and internal water pressure load finite element model

To calculate strain and displacement using FEA, a quarter-pipe model was developed which exploited the two planes of symmetry present in the experiment, as shown by Fig. 4(a). The simply-supported aspect of four-point bending was modelled by constraining the vertical movement (UY = 0) of a horizontal row of nodes at the pipe neutral axis (Fig. 4(b)). To approximate the point loads of four-point bending, the bending load was applied as a uniformly distributed pressure force over a small 2 × 2 mm patch (Fig. 4(c)). The internal water pressure load was applied to the inside faces of the specimen model as a uniformly distributed pressure (Fig. 4(d)). To account for the fact that the specimens were subject to an axial load caused by the internal water pressure reaction force on the hydraulic adaptors at each end of the specimen, a uniform pressure load with a negative magnitude was applied to the end face of the specimen model (Fig. 4(e)). A second-order hexahedral element mapped mesh with a 1 mm element size was used to discretise the specimen model. Mesh refinement confirmed that a 1 mm mesh size was sufficiently free from discretisation errors.

From the tensile tests reported by John et al. [6] it was known that the elastic modulus of the pipe specimen material could vary by about 10 GPa between specimens. Furthermore, the elastic modulus of spun GCI pipe specimens can vary depending on whether material from the inside or outside part of the pipe wall is sampled [6, 8]. The previously tested tensile specimens included a reduced gauge section that sampled the inside half of the pipe wall, whereas the specimens used in the verification experiments reported here did not feature a reduced gauge section. As a result, the elastic modulus determined from the tensile tests reported by John et al. [6] was thought unlikely to be representative of the specimens tested here.

The strain and displacement values calculated by the finite element model were very sensitive to the elastic modulus assigned to the elements. To prevent differences in the elastic moduli of the specimens used in the verification experiments from influencing the results of the validation exercise the elastic modulus was determined for each specimen and used in the finite element modelling. The elastic modulus of each of the two specimens was determined by iteratively altering the element elastic modulus in the 10 kN bending load finite element model until the calculated axial strain at the pipe invert was very close to the experimentally measured value. The remaining experimentally measured strain and displacement values were then available for the verification assessment. For consistency, the elastic modulus determined in this way for each specimen was also used to make estimations using the analytical approach. Using this approach reduced the uncertainty when comparing the measured and predicted strains and displacements, relative to if an average elastic modulus was assumed for both specimens.

### Trial Destructive Fatigue Test

To confirm that the experiment was able to cause fatigue failure of the GCI pipe specimens, three tests were run to failure with combined internal pressure and bending fatigue loading. So that these specimens would fail in the region of constant stress away from the load points a reduced gauge diameter was machined from the centre of the specimens, shown by Fig. 1(g). The fatigue mechanisms governing failure remain the same for failures within the high-cycle fatigue regime, so the trial destructive tests targeted failures between 10^3^ and 10^4^ load cycles. During each test an internal pressure amplitude around 3.8 MPa, with a load ratio of 0.12, and a bending load amplitude around 4.4 kN, with a load ratio of 0.1, were applied 180° out-of-phase to the pipe specimens. The exact loads were adjusted to account for slight variations in specimen geometry. Tests were suspended if failure did not occur after 10^5^ load cycles.

## Results

### Strain and Displacement Measurements and Estimates

This section contains results comparing the measured strains and displacements with the analytical and FEA estimates. The elastic moduli determined for the two specimens tested, and used in the estimate calculations, were 145 GPa and 135 GPa, respectively.

**Fig. 5:**
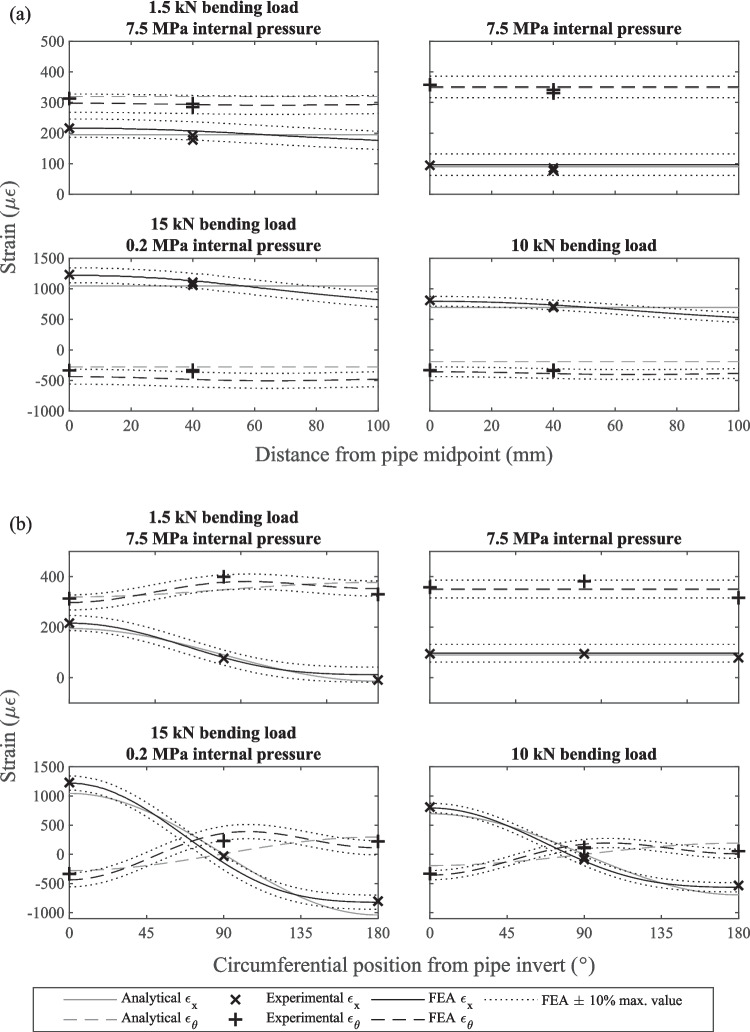
Measured and predicted strains (**a**) along the invert and (**b**) around the midpoint circumference of one of the specimens used for the strain and displacement validation experiments

**Fig. 6:**
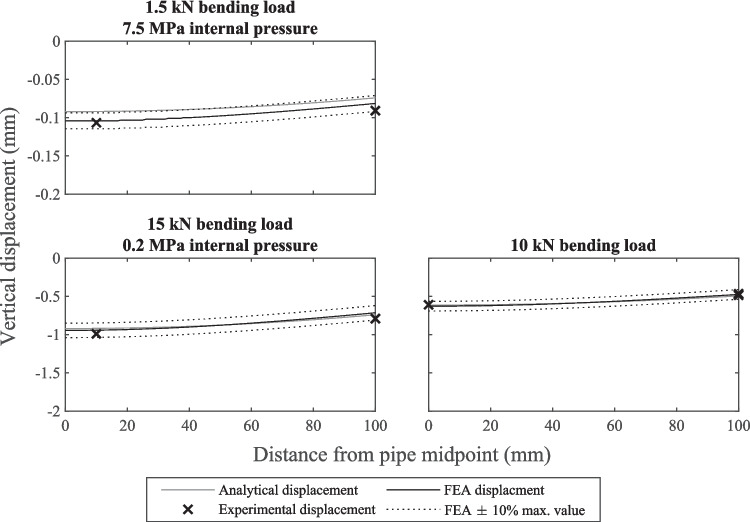
Measured and predicted vertical displacements along the invert of one of the specimens used for the strain and displacement validation experiments

The relationships observed between the measurements and estimations for the two specimens used for validation were very similar, so the results of just one specimen are presented here in graphical form. For that specimen, for each loading condition the measured and estimated strains along the specimen invert are shown in Fig. 5(a), and the measured and estimated strains around the circumference in the middle of the specimen are shown in Fig. 5(b). The measured and predicted vertical displacements along the specimen invert for the same specimen are shown in Fig. 6. Note that the vertical displacement measurements and predictions for the 7.5 MPa internal water pressure loading case are not provided as these were too small to measure reliably. To avoid penalising low magnitude strain readings the FEA prediction ± 10% bands were calculated as the predicted strain or displacement ± 10% of the maximum predicted strain or displacement.

All but three invert strain measurements across both specimens fell within ± 10% of the maximum predicted strain of the FEA predictions. The greatest invert strain error was 12.4% for a strain measurement of −330 µε and all invert strain measurements falling outside the ± 10% FEA prediction bands were located 40 mm away from the midpoint. All but one circumference strain measurement fell within ± 10% of the maximum predicted strain of the FEA predictions. The greatest circumference strain error was 10.8% for a strain measurement of 228 µε. Only two invert displacement measurements fell outside the ± 10% FEA prediction bands and these were both for the second pipe under the lowest bending load, meaning the measured displacement was very small (around −0.1 mm). The maximum error was 14.8%.

The invert strains measured 40 mm from the midpoint for each loading condition were nearly identical in each direction, indicating symmetric loading. The magnitude and distribution of the measured strains and displacements were also similar to the strains and displacements predicted using the classical beam theory and the thick-walled pressure vessel analytical solutions.

### Dynamic Load Effects

**Fig. 7:**
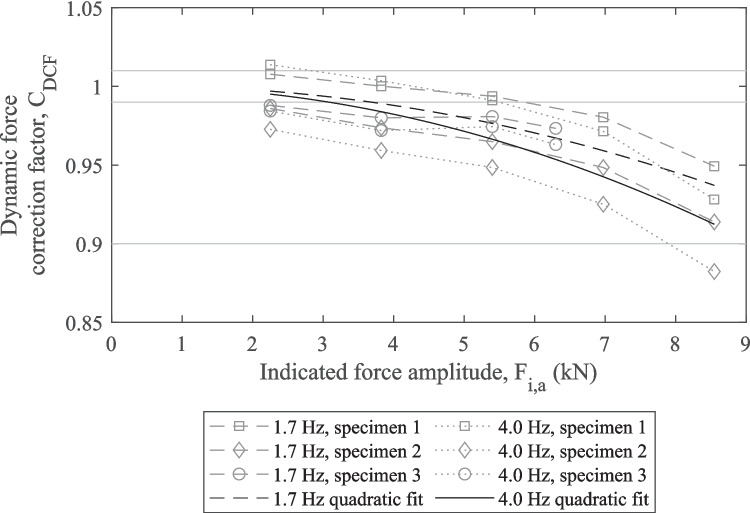
Plot of measured C_DCF_ values and calculated quadratic fits. Specimen 1 and 2 featured no gauge section reduction while specimen 3 had a reduced gauge section (horizontal grey lines indicate the ± 1% and −10% error limits)

The measured dynamic force correction coefficient (C_DCF_) values and calculated quadratic fits for 1.7 Hz and 4.0 Hz are plotted in Fig. 7. The results for each combination of specimen and frequency showed a non-linear decreasing relationship between F_dyn,a_ and C_DCF_ indicating that the dynamic force error increased with increasing force amplitude. For F_dyn,a_ ≤ 3.83 kN the C_DCF_ values were close to 1, whereas for F_dyn,a_ = 8.55 kN one C_DCF_ value fell below the 0.9 threshold. For both frequencies specimen 2 gave a greater dynamic force error than specimen 1. The dynamic force errors for specimen 3 fell between specimens 1 and 2, despite the fact specimen 3 had a reduced diameter gauge section giving it a lower stiffness. The quadratic fits, with forced y-intercept of 1, calculated for the 1.7 Hz and 4.0 Hz data were:

7 $${C}_{DCF,1.7Hz}=1+\left(7.93\times {10}^{-4}\right){F}_{i,a}-\left(9.54\times {10}^{-4}\right){F}_{i,a}^{2}$$

and

8 $${C}_{DCF,4Hz}=1+\left(6.89\times {10}^{-4}\right){F}_{i,a}-\left(1.28\times {10}^{-3}\right){F}_{i,a}^{2}$$

These quadratic fits provided good approximations of the non-linear trend observed for each specimen, however, due to the scattering of the observations the R^2^ values were low at 0.67 and 0.64, respectively. The quadratic fits fell within the ± 10% error threshold and show that on average the dynamic force error was greater at 4.0 Hz than at 1.7 Hz.

**Fig. 8:**
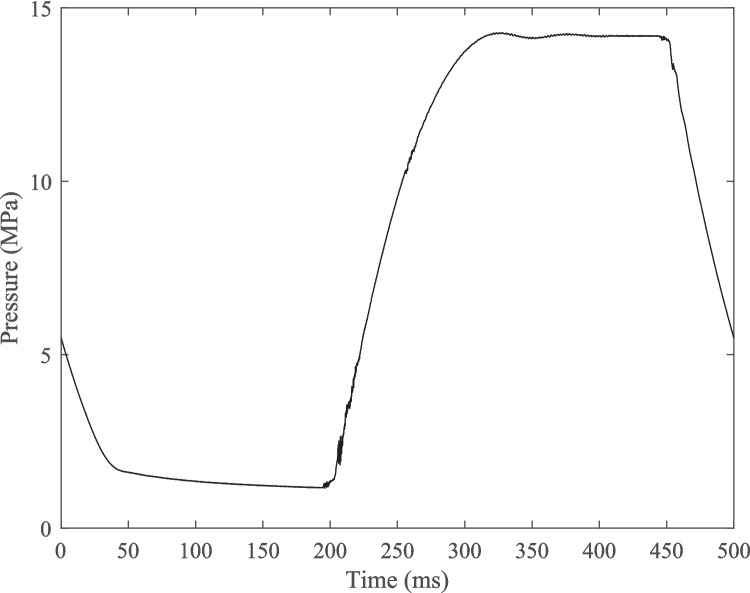
Plot of a single 2 Hz internal water pressure cycle recorded at a 10 kHz sampling rate

Figure 8 shows a representative 2 Hz loading frequency, 6.55 MPa amplitude internal water pressure cycle measured at a 10 kHz sampling rate. Several very high-frequency pressure instabilities with an amplitude of about 0.41 MPa occurred at the start of the pressure increase (at about 210 ms in Fig. 8), likely caused by the sudden opening of the solenoid valve. A small amount of overshoot occurred as the maximum pressure was reached (at about 300 ms in Fig. 8) resulting in a high-frequency oscillation with a maximum amplitude of 0.08 MPa. The maximum amplitude of a sub-cycle was therefore about 6.3% of the main cycle amplitude.

### Cyclic Loading Accuracy

**Fig. 9:**
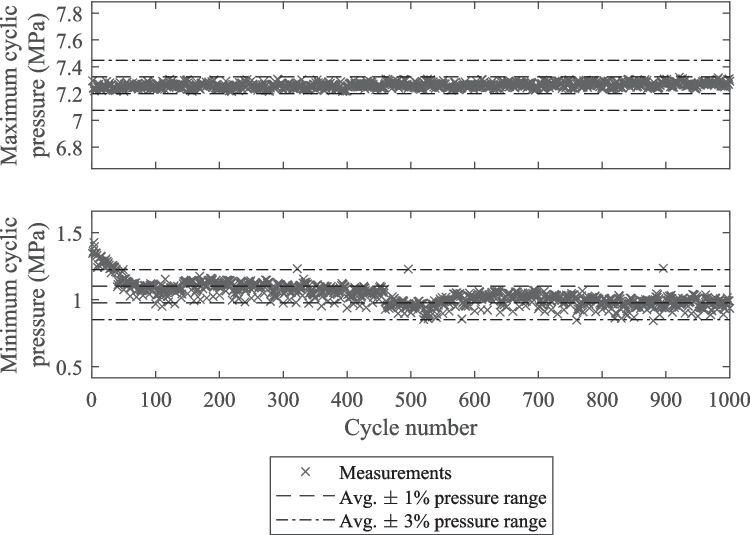
Plot of measured maximum and minimum cyclic pressures for internal water pressure loading over 10.^3^ cycles for test 3 from Table 2

The percentage of cycles from each internal water pressure load accuracy test that had amplitudes within error thresholds of ± 1%, ± 3%, and ± 5% of the average test amplitude are given in Table 2. For tests 1 and 2 all cycles had amplitudes within ± 3% of the average, however, for test 3 95.3% of cycles fell within this range. Figure 9 shows the minimum and maximum cyclic pressures for test 3. The accuracy of the maximum pressure values for test 3 were very good, all falling within ± 1% of the average. During the first 100 pressure cycles of test 3 the minimum cyclic pressures gradually stabilised. The final row of Table 2 gives the amplitude error for test 3 excluding the cycles before the hundredth, from which it can be seen that the percentage of cycles with an amplitude error within ± 3% was 99.6%.

**Table 2:** Percentage of internal water pressure cycles with amplitudes falling within ± X% of the average during 10^3^ cycle duration trial tests

Test	Pressure cycle amplitude error		
	$\left|{P}_{a,err}\right|\le 1\%$	$\left|{P}_{a,err}\right|\le 3\%$	$\left|{P}_{a,err}\right|\le 5\%$
1	34.4%	100.0%	100.0%
2	97.6%	100.0%	100.0%
3	52.2%	95.3%	99.3%
3 (cycles 100 to 1,000)	57.7%	99.6%	100.0%

**Fig. 10:**
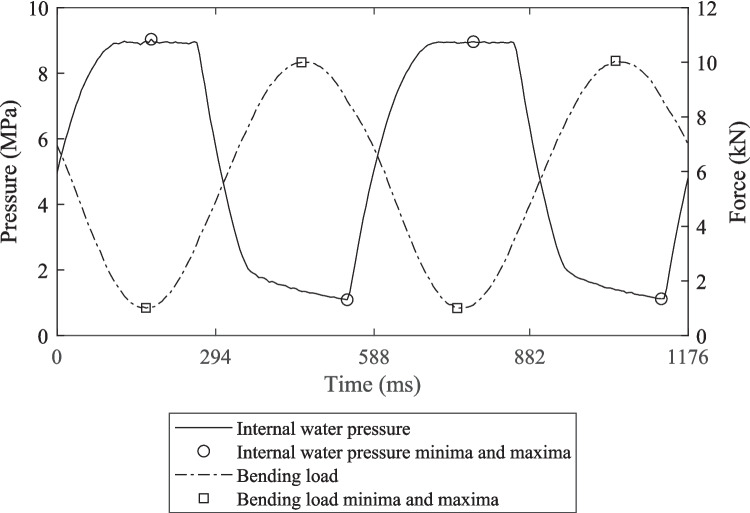
Plot of measured internal water pressure and bending load for 180° out-of-phase loading during test 2 from Table 3

An example of a representative 180° out-of-phase internal pressure and bending loading time-series is given in Fig. 10. The internal water pressure load remained close to its maximum value for about 140 ms, meaning the minimum bending load consistently occurred while the pressure load was at its maximum. The minimum internal pressure load occurred instantaneously just before the pressure suddenly increased, making it difficult to align the maximum bending load and minimum internal pressure. To give consistent performance, the phasing was set so that the maximum bending load occurred before the minimum pressure load where the rate of change of internal pressure was relatively low.

**Table 3:** Percentage of pressure cycles, defined using the locations of minimum and maximum cyclic bending load, during 180° out-of-phase bending and internal pressure loading with amplitudes ≥ X% of the average during 10^3^ cycle duration trial tests

Test	Average true pressure amplitude (MPa)	Pressure cycle amplitude error		
		${P}_{a,err}\ge -1\%$	${P}_{a,err}\ge -3\%$	${P}_{a,err}\ge -6\%$
1	2.62	0.0%	0.4%	98.8%
2	3.93	0.0%	5.5%	100.0%
3	4.92	0.1%	96.6%	100.0%

**Fig. 11:**
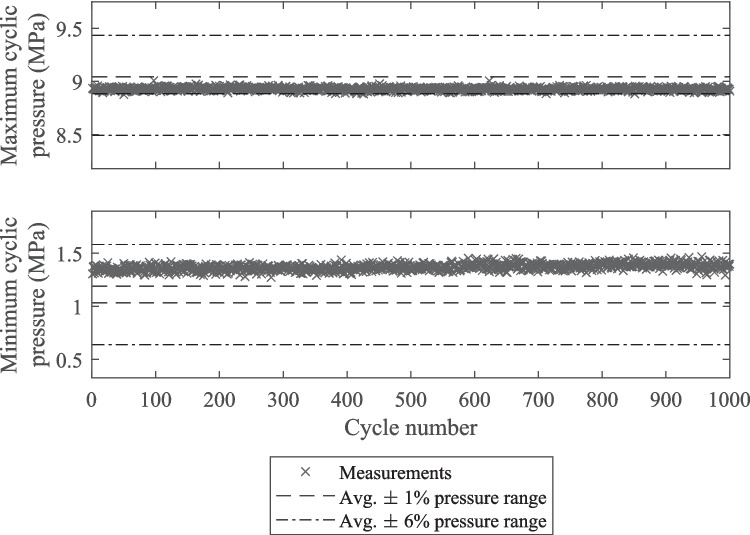
Plot of measured cyclic pressures during 180° out-of-phase bending and internal pressure loading defined using the location of maximum and minimum bending loads over 10.^3^ cycles for test 2 from Table 3

Figure 11 shows the minimum and maximum cyclic pressures for test 2, which were representative of the trends observed in the other two tests. It can be seen from Fig. 11 that the chosen load phasing gave maximum cyclic pressures, defined at the minimum cyclic bending load, within ± 1% of the true value. The minimum pressure, defined at the maximum cyclic bending load, was consistently above the true minimum cyclic pressure, however, the degree of overestimation was stable. The percentage of cycles from each test which had internal pressure amplitudes, defined by the pressures occurring at the minimum and maximum bending loads, within error thresholds of ≥ −1%, ≥ −3%, and ≥ −6% of the average true cyclic pressure amplitude are given in Table 3. Due to the slight misalignment of the maximum bending load and minimum internal pressure very few pressure cycles from the three tests had pressure amplitudes within ± 1% of the true pressure amplitude. For tests 1 and 2, most pressure cycle amplitudes fell between −3% and −6%, whereas for test 2 most errors fell between −1% and −3%. Comparing the three tests reported in Table 3 shows that the amplitude error was greater at lower true pressure amplitudes. This was because the difference between the true minimum cyclic pressure values and pressure values at the maximum cyclic bending load were similar for all pressure amplitudes. At higher pressure amplitudes this difference in minimum pressures represented a smaller proportion of the total amplitude.

### Trial Destructive Fatigue Test

**Fig. 12:**
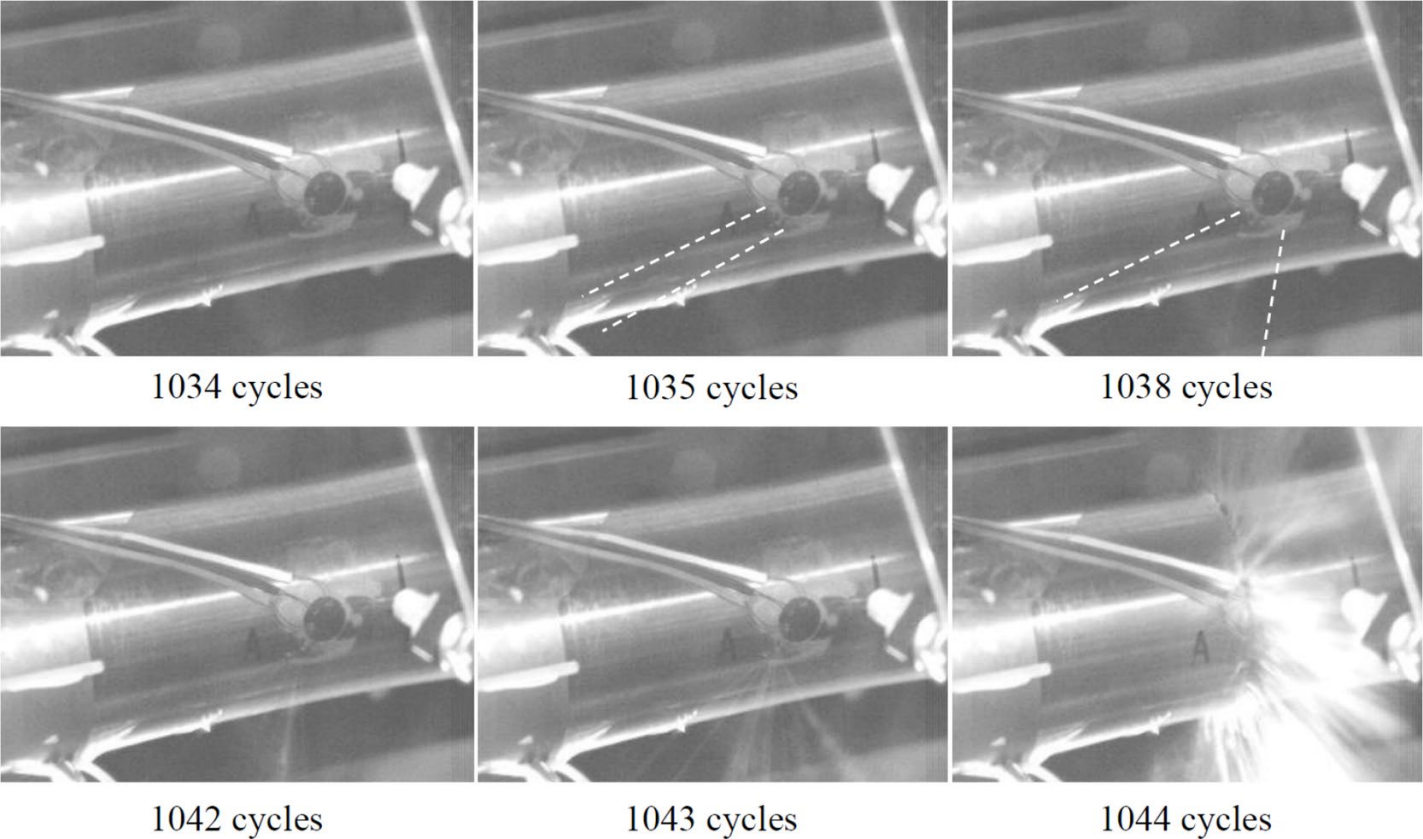
High-speed video stills showing the evolution of a leak at the invert of a pipe specimen prior to failure under 180° out-of-phase internal water pressure and bending loading (white dashed lines indicate the position of the water jet where this is faint)

Of the three fatigue tests of uniform wall-loss pipe specimens, two pipes developed a leaking crack after 1,035 and 5,900 load cycles then split in two via a full circumferential crack after a further 9 and 19 load cycles, respectively, and one pipe survived 10^5^ load cycles without developing a leak. Figure 12 shows stills from the high-speed camera footage of one test illustrating: the first appearance of a leaking crack after 1,035 cycles, the development of the leak over the following 8 load cycles, and the sudden formation of a full circumferential crack after 1,044 load cycles.

The fracture surfaces of these pipes were visually examined but an oxide layer had formed on the fracture surface due to wetting during the failure process. As a result, it was not possible to discern any features of interest from the fracture surface. Other than the oxide layer, the fracture surfaces were visually very similar to those reported by John et al. [6] for uniaxial fatigue tests of the same material, indicating that axial stresses controlled the final fast fracture process in the biaxial fatigue tests reported here.

## Discussion

### Stresses Resulting from the Applied Loads

To determine whether specimens experienced the desired stresses when bending and/or internal pressure loading was applied, experimental strain measurements under known loads were compared with predictions made using analytical solutions and FEA, both of which assumed linear-elastic behaviour. The relatively linear stress–strain behaviour of the specimen material up to failure, previously reported by John et al. [6], meant that assuming linear-elastic behaviour of the material was reasonable and hence good strain agreement equated to good stress agreement.

The four-point bending equipment developed was able to load pipe specimens in a way that was very close to ideal four-point bending according to the classical beam theory solution, although, deformation of the pipe cross-section meant that the experimental strains were closer to the FEA estimations than the analytical estimations. Deformation of the pipe cross-section is evidenced by disagreement between the analytical solution and the strains measured around the pipe circumference, shown by Fig. 5(b). The finite element model boundary conditions, which can be assumed to reflect the experiment boundary conditions, included a pinned joint at the specimens' geometric centroid that was free to rotate and translate, except in the vertical direction (see Fig. 4(b)), which is very similar to the simply supported condition.

The finite element model of the specimens assumed linear stress–strain behaviour and matched the experimental strain and displacement measurements for a range of bending loads representative of those which would cause fatigue failure, implying that the physical specimens also demonstrated linear stress–strain behaviour. This shows that when analysing GCI pipes subject to bending loads of a magnitude relevant to high-cycle fatigue the stress–strain non-linearity corrections employed by Seica and Packer [15] for static failure analysis are unnecessary, which simplifies the analysis procedure.

The strains experienced by pipe specimens subject to only an internal pressure load agreed well with the closed-ends thick-walled pressure vessel relation (equations (5) and (6)) and FEA estimations, as shown by Fig. 5. The FEA model used the same boundary conditions for combined loading and bending-only loading. The good agreement between the experimental strains and FEA estimated strains for both of these loading conditions shows that the application of internal pressure loading did not influence the way in which the specimens interacted with the load points and supports. Therefore, superposition could be used to determine the stresses experienced by specimens under combined loading.

In summary, the good agreement between the experimental strain and displacement measurements and the strains and displacements predicted using FEA meant that the four-point bending equipment created stress–strain conditions very similar to the idealised four-point bending and closed-end pressure vessel scenarios, with the exception of some deformation of the specimen cross section during bending. A limitation of the closed-ended pressure vessel arrangement was that hoop stress caused by internal water pressure loading could not be applied to a pipe independently due to the coupled in-phase axial stress caused by the end caps. This did not represent a significant limitation given that the intended function of this experiment was to investigate biaxial fatigue loading.

### Dynamic Load Effects

The dynamic force correction factor was determined for the full load range at both 1.7 and 4 Hz according to ASTM E467-21 [32] and BS ISO 4965–1 [33]. Dynamic force errors did occur, particularly at higher load amplitudes due to the relatively large displacements of the bending load actuator (up to about 2 mm), however every measurement but one was within the recommended ± 10% tolerance, as shown by Fig. 7. Neither the average value at each load or the fitted curves exceeded the ± 10% tolerance so the dynamic force correction factors given by equations (7) and (8) could be used to correct the applied bending forces.

No significant dynamic load effects were detected during high-frequency recording of pressure cycles, as shown by Fig. 8. As a result, defining and counting pressure cycles by their minimum and maximum values was representative of the loading applied to specimens.

### Cyclic Loading Accuracy

Cyclic control of the bending load was excellent, as expected due to the use of a commercial system, with all peak and valley loads for the 10^3^ cycles tested lying within ± 1% of the load range, as recommended by ASTM E467-21 [32] and BS ISO 4965–1 [33]. No start-up transient behaviour was detected.

For internal pressure loading, transient start-up behaviour was detected (see Fig. 9) but this was not problematic as it resulted in slightly lower pressure amplitudes for about 100 cycles and no load spikes. Peak pressure errors complied with the standards whereas valley pressure errors were within ± 3% of the pressure range (see Table 2). The corresponding ± 3% pressure amplitude error was deemed to be acceptable.

For biaxial loading, consistent load phasing close to 180° out-of-phase was achieved, with the difference unlikely to significantly influence the fatigue results obtained. Very good alignment of the maximum pressure and minimum bending load was achieved, however the shape of the internal water pressure waveform meant that the pressure at the maximum bending load was up to 6% of the pressure range greater than the minimum (see Table 3 and Figs. 10 and 11).

### Trial Destructive Fatigue Test

The trial destructive fatigue tests show that the experiment presented in this paper can cause fatigue failures of small-diameter GCI pipes specimens within the high-cycle fatigue regime using 180° out-of-phase bending and internal pressure loading. As a result, the experiment is suitable for investigating the type of fatigue failures proposed by Brevis et al. [16] and Jiang et al. [17]. The fact that one pipe survived 10^5^ load cycles highlights the inherent variability of GCI pipe material properties while also proving the ability of the experiment to run long duration fatigue tests.

Using high-speed camera footage, leaks were detected in the pipe specimens prior to bursting (see Fig. 12). These leaks persisted for less than 1% of the total cycles-to-burst, indicating that in-service GCI pipes in similar conditions are unlikely to experience prolonged periods of leakage and will instead burst with limited warning.

### Significance

The trial destructive fatigue tests revealed the leakage behaviour of fatigue cracks formed in GCI pipes with uniform wall-loss subject to combined internal pressure and bending fatigue loads for the first time. The very short duration of stable leakage in these tests show that in-service GCI pipes in similar conditions are less likely to be responsible for long-term, stable leaks, but instead may burst with little warning. Therefore, these results can be used to help identify GCI water pipes that are at risk of developing sudden burst failures.

The novel biaxial fatigue experiment developed and tested for this study enables future investigations to obtain fatigue data for notched GCI pipe specimens subject to multiaxial fatigue stresses. This data is essential for the development and validation of a multiaxial notch fatigue model that can be used to predict fatigue failures of in-service GCI water pipes, and hence enable informed pipe replacement decisions to be made to reduce the water lost via leaks and bursts.

The primary motivation behind the development of this novel out-of-phase biaxial loading fatigue experiment for GCI pipe specimens was that previous GCI material test results indicate that this type of loading may result in more rapid fatigue damage accumulation, as discussed in Sect. 1. The damaging effect of out-of-phase biaxial fatigue loading relative to other fatigue loads is determined by the fatigue crack growth mechanism, which can vary significantly between materials [37]. As a result, it may be beneficial to use the novel experimental methodology presented in this paper to explore the response of other pipe materials to out-of-phase biaxial fatigue loading if preliminary coupon tests indicate that the material may accumulate fatigue damage more rapidly under this type of loading. Note that while coupon tests can provide an indication of the pipe material’s fatigue behaviour, pipe specimen tests are needed to capture pipe specific failure behaviours and the impact of irregular features such as weld seams, corrosion damage, and joints.

While this fatigue testing facility was developed for GCI water pipes it could be deployed, with minimal modifications, to investigate the out-of-phase biaxial loading fatigue performance of other pipe materials and pipe joining techniques, provided pipe specimens with diameters less than 100 mm can be sourced. For example, future work could utilise this facility to investigate the fatigue behaviour of PVC water pipes, which are also reported to have high failure rates, are susceptible to longitudinal fatigue cracking, and frequently fail at joints [3]. As a result, this testing capability enables new avenues of research to quantify the mechanical performance of existing water distribution networks and ensure the robustness of future water distribution networks. Furthermore, the fatigue response of pipelines to cyclic internal pressure and bending loads in applications such as oil and gas transmission, process industries, and power generation has received frequent research interest [24–26, 28]. As a result, it may be beneficial to investigate SS316 and other pipe materials common to these applications under out-of-phase biaxial fatigue loading if preliminary investigations show this loading may be of concern. Lastly, while this experiment was developed for high-cycle fatigue testing, selecting appropriate loads and specimen dimensions would enable low-cycle fatigue tests to be performed.

### Summary

The novel biaxial fatigue experiment was able to consistently apply bending and internal water pressure fatigue loading, either independently or together with a 180° phase difference, resulting in specimens experiencing a stress–strain state very similar to the ideal conditions. The major source of load error was the dynamic load effect resulting from acceleration and deceleration of the bending load points, however, this was quantified and accounted for in the fatigue test result processing. Trial tests have shown that this experiment can generate high-cycle fatigue failures in small-diameter GCI pipes. The experiment developed for this project was unique because it was able to apply both internal water pressure and bending fatigue loading with a 180° phase difference to pipe specimens. The experiment also enables extensive fatigue testing programmes through its high loading frequency (achieving $5\times {10}^{4}$ cycles per 8-h working day). Given the importance of destructive testing of pressurised pipes to the water industry and other sectors, such as oil and gas transmission, process industries, and power generation, the experiment developed for this project has a wide range of potential applications.

## Conclusions

To enable extensive investigations into the fatigue failure mechanism of GCI water pipes for the first time, the work presented in this paper aimed to develop and test a novel experiment capable of causing controlled fatigue failures of GCI pipe specimens in the high-cycle fatigue regime using combined bending and internal water pressure fatigue loading. The conclusions reached are as follows:

- The experiment developed was able to apply combined, out-of-phase internal pressure and bending fatigue loads accurately and consistently to small-dimeter GCI pipes, and cause these pipes to develop high-cycle fatigue regime failures.
- The non-linear compressive stress strain behaviour of GCI pipes can be neglected for stress analysis of GCI pipes subject to bending loads of a magnitude that will cause high-cycle fatigue failure, significantly simplifying the analysis.
- The lifespan of leaking fatigue cracks in GCI pipes with uniform wall-loss subject to combined internal pressure and bending fatigue loads is less than 1% of the total cycles-to-burst. As a result, in-service GCI pipes in similar conditions are less likely to be responsible for long-term, stable leaks, and are instead likely to burst with limited warning.

The novel experiment presented in this paper will be used to conduct further research into the interaction between corrosion pitting and the fatigue loads applied to GCI pipes. It is intended that this work will ultimately contribute to reducing leakage water loss by enabling better-informed condition assessment of corroded in-service GCI water pipes. The experimental methodology presented here is also suitable for investigating other pipe materials where out-of-phase biaxial loading is likely to accelerate fatigue crack growth.

## Data Availability

The original contributions presented in the study are included in the article; further inquiries can be directed to the corresponding author.

## Nomenclatures

${a}_{b}$: Distance along a beam between a support and the nearest load point
${C}_{DCF}$: Dynamic force correction factor
$$E$$: Elastic modulus
$${F}_{b}$$: Bending load
${F}_{i,a}$: Indicated force amplitude
$I$: Second moment of area
${L}_{b}$: Distance along a beam between the two supports
${P}_{a,err}$: Pressure amplitude error
$${P}_{i}$$: Internal pressure
$${r}_{e}$$: External radius
$${r}_{i}$$: Internal radius
$x$: Axial/longitudinal position
$$y$$: Vertical position
$\Delta {F}_{dyn}$: True dynamic force range
$\Delta {F}_{i}$: Indicated force range
$$\delta y$$: Vertical deflection
$${\varepsilon }_{x}$$: Axial strain
$${\varepsilon }_{\theta }$$: Hoop strain
$$\nu$$: Poisson ratio
